# A Near-Peer Mentorship Program that Sustains a Student-Faculty Partnership Co-creating Curriculum-Aligned Formative Multiple-Choice Questions in Preclinical Medical Education

**DOI:** 10.1007/s40670-025-02406-8

**Published:** 2025-05-07

**Authors:** Laura M. Banks, Jason L. Hirsch, Jarod Karom, Corinne Stanforth, Erryk S. Katayama, Lin Abigail Tan, Matthew C. Reslink, Melissa M. Quinn, Christopher R. Pierson

**Affiliations:** 1 The Ohio State University College of Medicine, Columbus, OH 43210 USA; 2 The Ohio State University Department of Biomedical Education and Anatomy, Division of Anatomy, Columbus, OH 43210 USA; 3 The Ohio State University Department of Pathology, Columbus, OH 43210 USA; 4 Department of Pathology and Laboratory Medicine, Nationwide Children’s Hospital, Columbus, OH 43205 USA

**Keywords:** Active learning, Co-creation, Formative practice, Near-peer mentorship, Multiple-choice question

## Abstract

Professor-Reviewed Exam Practice (PREP) is a student-faculty partnership that co-creates curriculum-aligned formative questions for the entire preclinical medical school class to use in active learning. PREP has sustained its productivity over three medical school classes. Here we describe PREP’s recruitment process and its near-peer mentorship program that prepares new members to independently create PREP-style formative questions. PREP’s productivity and the utilization of its formative questions across classes is evaluated, demonstrating that the workflow is sustainable. We believe that this program could be adopted by other schools to broaden the availability of free curriculum-aligned formative questions in preclinical medical education.

**Supplementary Information:**

The online version contains supplementary material available at 10.1007/s40670-025-02406-8.

## Background

Medical knowledge is often evaluated in preclinical undergraduate medical education using summative assessments commonly comprised of multiple-choice questions (MCQs) [1]. As a result, students value formative MCQ (FMCQs) to practice applying medical knowledge prior to high stakes summative assessments [1–3]. Faculty value FMCQs because they promote active learning. Despite their value to learning and teaching, the supply of FMCQs is often limited [2–6]. In response, students turn to outside resources; however, they tend to be expensive, which may lead to inequities. These resources are also not specific to any particular school’s curriculum, potentially rendering them ineffective tools for student preparation given each school’s summative assessments are unique. To meet the need for high-quality, free FMCQs that align with our school’s curriculum, we founded Professor-Reviewed Exam Practice (PREP). PREP is a student-faculty partnership consisting of a team of preclinical medical students who write vignette-style, single-best response FMCQs with feedback covering each lecture in the preclinical curriculum that are reviewed by faculty prior to distribution to the class [7]. We reported on PREP’s workflow and initial outcomes and now expand on it sustainability [7].

When members of the first PREP team completed the preclinical curriculum and progressed to clinical clerkships, it posed a challenge: how would PREP sustain its high level of success and standards going forward? Devising a sustainable system was necessary to set expectations, support new members, and prevent recycling of FMCQs from year-to-year, ensuring the FMCQs remained up-to-date and relevant as the curriculum changed over time. In response, we developed an application process for potential PREP members for the first-year (M1) students in the fall semester and coupled it to a near-peer mentorship program where second-year M2 (M2) PREP members served as mentors. We choose this model based upon the known benefits of near-peer mentorship in promoting professional and personal development and aiding transitions in M1 students [8].

## Activity

At the start of the M1 academic year, an informational meeting was held with potential applicants to describe PREP’s expectations, which include committing 5 to 10 h weekly to creating 3 to 5 unique questions and explanations, and reviewing 12 to 20 questions constructed by other members. A PREP application was generated using Google Forms (Alphabet Inc, Googleplex, Mountain View, CA) and distributed to the M1 students via campus email (Supplemental Information). The application was designed to assess the interest level, commitment, and relevant background of each applicant. The application asked students to write a FMCQ from the current curricular unit with feedback, revise an intentionally suboptimal sample FMCQ provided by PREP, explain their reasons for joining PREP, and share past teaching experiences. Applications were collected and anonymously rated by all seven members of the M2 PREP team according to the quality of submitted FMCQs, which were assessed on accuracy, relevance, the effectiveness of constructive feedback, extent of prior teaching experience, and enthusiasm for membership.

Fifteen applications were received from the class of 2027 PREP team, which was an increase by six over the first year of recruitment (class of 2026). Emphasis in application review was placed on motivation for joining PREP and prior teaching experience. Intrinsic motivation was considered crucial to the PREP’s success due to the volume of work and deadlines. Prior teaching experience was valued as a reflection of an applicant’s commitment to service through education. It was believed that PREP-style question-writing could be learned and practiced within the mentorship program, making this a secondary priority. Reviewed applications were assigned a score ranging from 1 to 10, with a score of 10 considered as exceptional, 5 as average, and 1 as unsatisfactory. The seven highest scores were accepted. The overall average application score was 7.7 (range, 6.3–9.7), with an average successful application score of 8.4 (range, 7.6–9.7).

Each new M1 member was paired with a M2 mentor for one-on-one instruction and feedback regarding the FMCQ writing process prior to mentees submitting FMCQs for class use. Instructional videos were created and supplemented mentor instruction by detailing the process of FMCQ creation and distribution. Mentees were shown how to identify high-yield information pertaining to the lecture learning objectives and implement information into a clinical scenario captured by a vignette-style, single-best response FMCQ. Mentees were instructed that effective vignettes provide adequate information, including pertinent positive and negative findings to enable the reader to distinguish the appropriate response from the distractors. Response choices were standardized to be a few words in length. Plausible incorrect distractors were used whenever possible to mimic the format of summative assessments at our school and by the National Board of Medical Examiners.

The perceived effectiveness of PREP near-peer mentor program was evaluated using voluntary, anonymous REDCap surveys [9] that were sent to mentees and mentors (Supplemental Information) to capture both perspectives. Specifically, the survey assessed the mentee-mentor meetings, instructional videos, and overall mentorship quality.

## Results and Discussion

**Fig. 1:**
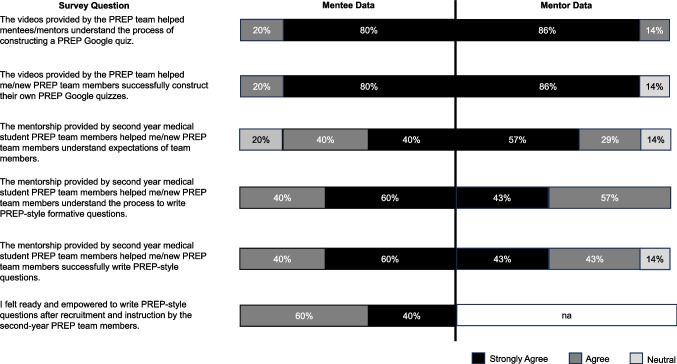
Results for each survey question from both mentors and mentees. Survey questions were written to facilitate direct comparison of mentee and mentor perspectives. Likert Scale ranged from “Strongly Agree” to “Strongly Disagree.” Both “Disagree” and “Strongly Disagree” were never chosen and are therefore not included (na, not applicable). Figure created with Microsoft PowerPoint

Twelve of 14 PREP members responded to the anonymous survey (86% overall response rate), which included all seven mentors (100% response rate), and 5 of 7 mentees (71% response rate) (Fig. 1). All mentees (100%) agreed or strongly agreed that the videos were beneficial to their understanding of PREP’s processes and FMCQ writing. Mentees’ perceptions of their mentor–mentee relationship were positive in terms of aiding their understanding of expectations (80% strongly agreed or agreed), the FMCQ writing process (100% strongly agreed or agreed), and the empowerment they felt (100% strongly agreed or agreed) from the mentor–mentee relationship. Mentors’ perceptions of the mentor–mentee relationship were also positive. All mentors (100% strongly agreed or agreed) believed that the videos aided mentee understanding of PREP processes and 86% strongly agreed or agreed that they helped their mentees write PREP-style FMCQ. Mentors considered the mentee-mentor pairings successful in terms of enhancing mentee understanding expectations (86% strongly agreed or agreed), communicating the process of writing PREP-style FMCQs (100% strongly agreed or agreed), and allowing their mentee to successfully write PREP-style FMCQs (86% strongly agreed or agreed). Mentees from PREP team members in the classes of 2026 and 2027 were asked to rate the assistance from M2 mentors and PREP mentors were asked to rate their own mentorship using a 1 to 10 scale. On average, mentors rated their own mentorship as 8.1/10 (range, 6–10); mentees rated the mentorship received as 9.2/10 on average (range, 8–10), indicating alignment between mentee and mentor perceptions of the relationship and its effectiveness.

**Fig. 2:**
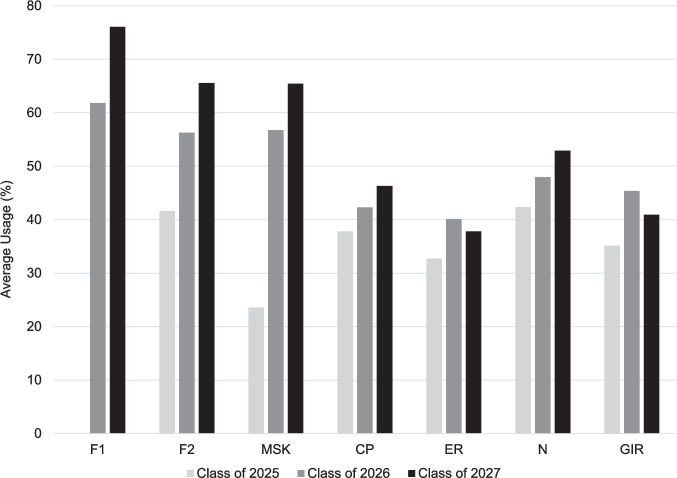
Year-to-year class participation counts for PREP unit assessments. The inaugural PREP team, the Class of 2025, started tracking participation during the first full unit of question creation, Foundations 2 (F2) that teaches basic pathology, genetics, inflammation, and immunology. Average percent usage was calculated by dividing the average number of submissions per unit by the class size, 209. Abbreviations: F1, Foundations 1 Block; F2, Foundations 2 Block; BM, Bone and Muscle Disorders Block; CP, Cardiopulmonary Disorders Block; ER, Endocrine-Reproductive Disorders Block; N, Neurological Disorders Block; GIR, Gastrointestinal-Renal Disorders Block. Figure created with Microsoft Excel and PowerPoint

The near-peer mentorship program has sustained PREP’s output. Over the last 3 years, PREP generated 2662 unique questions spread over 137 assessments that were distributed by Google Forms to the class on a weekly basis (Table 1). This approaches the size of some popular commercial question-banks. Out of these 2662 questions, the first PREP team (Class of 2025) created 828 questions, while the second PREP team (Class of 2026) contributed 1038 questions. Our latest cohort to finish the curriculum, the Class of 2027, created 796 questions. The quality of question-writing was sustained throughout each class by consistent faculty review, and the quantity of questions varied depending on each PREP cohort’s discretion and changes to the curriculum. PREP FMCQ usage by the class body was crucial to understanding if PREP met its objectives and if its FMCQs were valued. FMCQ usage by each class overall mostly increased over the last 3 years in each curricular block that PREP deployed FMCQs (Fig. 2). The average usage rate of PREP FMCQs increased from 35% for the Class of 2025, 50% for the Class of 2026, and up to 55% for the Class of 2027. Within each cohort, participation was highest in early M1 year as students likely sampled multiple study resources before selecting resources to utilize on an ongoing basis. When students from each cohort progressed through M2 year, there was a downtrend in participation (Fig. 2) as students likely shifted focus from studying primarily for institution exams to preparing for USMLE Step 1 via third-party resources. This participation trend was consistent between all three cohorts (Fig. 2). These reported rates have limitations, since students may access PREP FMCQs without submitting the form or students may submit multiple forms. The survey data was positive overall but the small size of the PREP team likely limits the scope of free-text comments collected.

**Table 1:** PREP productivity: total number of formative multiple-choice questions generated in each curricular block by all three PREP teams

Curricular unit	Class of 2025 PREP team	Class of 2026 PREP team	Class of 2027 PREP team
Foundations 1	17^	63	66
Foundations 2	76	91	98
Bone & Muscle	89	139	100
Cardiopulmonary	230	262	200
Endocrine & Reproductive	148	163	136
Neurological	158	186	116
Gastrointestinal & Renal	110	134	80
**Total**	828	1,038	796

^The PREP workflow was piloted in this block, which reflects the low number of FMCQs

While some student groups provide peer tutors or teaching assistants, PREP offers a unique approach to promoting active learning by creating a student-faculty partnership to write FMCQs for the benefit of the class as a whole. The demanding pace and nature of the preclinical curriculum posed challenges that the application process, instructional videos, and near-peer mentorship have successfully managed. PREP’s productivity in terms of total FMCQs generated and class FMCQ usage rates over the last 3 years along with the positive perceptions of mentees and mentors alike for the near-peer mentorship program suggest that the PREP workflow and mentorship model are successful and sustainable. We believe that other institutions could adopt this near-peer mentorship model to build a similar student-faculty partnership to foster affordable, curriculum-specific active learning in their preclinical curriculum.

## Supplementary Information

Below is the link to the electronic supplementary material.

ESM 1 (PDF 121 KB)
