# Supplementary material for: A Near-Peer Mentorship Program that Sustains a Student-Faculty Partnership Co-creating Curriculum-Aligned Formative Multiple-Choice Questions in Preclinical Medical Education
Source: Med Sci Educ. 2025 May 7;35(4):1921–5. doi: 10.1007/s40670-025-02406-8 (PMC12532483; doi:10.1007/s40670-025-02406-8)
Supplement: Supplementary file 1 — (PDF 121 KB) [file 40670_2025_2406_MOESM1_ESM.pdf]

A near-peer mentorship program that sustains a student-led, faculty-guided partnership co-creating curriculum-aligned formative multiple-choice questions in preclinical medical education

Medical Science Educator

Laura M. Banks[1,*], Jason L. Hirsch [1,*], Jarod Karom[1], Corinne Stanforth [1], Erryk S. Katayama[1], Lin Abigail Tan[1], Matthew C. Reslink[1], Melissa M. Quinn[2], Christopher R. Pierson[2, 3, 4]

1, The Ohio State University College of Medicine, Columbus, OH, 43210, USA

2, The Ohio State University Department of Biomedical Education and Anatomy, Division of Anatomy, Columbus, OH, 43210, USA

3, The Ohio State University Department of Pathology, Columbus, OH, 43210, USA

4, Nationwide Children's Hospital, Department of Pathology and Laboratory Medicine, Columbus, OH, 43205, USA

*, Contributed equally

Corresponding author

Christopher R. Pierson, MD, PhD

Nationwide Children's Hospital

Department of Pathology and Laboratory Medicine, J0359

700 Children's Drive

Columbus, OH

P: 614.722.5450

Christopher.Pierson@nationwidechildrens.org

ORCID: 0000-0002-5553-3096

Mentee Survey	
**Questions About Constructing the PREP Google Quiz**	
The videos provided by the PREP team helped me understand the process of constructing a PREP Google Quiz.	( ) Strongly Disagree<br>( ) Disagree<br>( ) Neutral<br>( ) Agree<br>( ) Strongly Agree
The videos provided by the PREP team helped me successfully construct my own PREP Google quizzes.	( ) Strongly Disagree<br>( ) Disagree<br>( ) Neutral<br>( ) Agree<br>( ) Strongly Agree
**Questions About the Mentor-Mentee Relationship**	
The mentorship provided by second year medical student PREP team members helped me understand expectations of team members.	( ) Strongly Disagree<br>( ) Disagree<br>( ) Neutral<br>( ) Agree<br>( ) Strongly Agree
The mentorship provided by second year medical student PREP team members helped me understand the process to write PREP-style formative questions.	( ) Strongly Disagree<br>( ) Disagree<br>( ) Neutral<br>( ) Agree<br>( ) Strongly Agree
The mentorship provided by second year medical student PREP team members helped me successfully write PREP-style questions.	( ) Strongly Disagree<br>( ) Disagree<br>( ) Neutral<br>( ) Agree<br>( ) Strongly Agree
**Questions About All Resources**	
I felt ready and empowered to write PREP-style questions after recruitment and instruction by the second-year PREP team members.	( ) Strongly Disagree<br>( ) Disagree<br>( ) Neutral<br>( ) Agree<br>( ) Strongly Agree
Overall, I would rate (1-10 scale) the help provided by the second year PREP team members as:	( ) 1 (lowest quality)<br>( ) 2<br>( ) 3<br>( ) 4<br>( ) 5<br>( ) 6<br>( ) 7<br>( ) 8<br>( ) 9<br>( ) 10 (highest quality)

Mentor Survey	
**Questions About Constructing the PREP Google Quiz**	
The videos provided by the PREP team helped me understand the process of constructing a PREP Google Quiz.	( ) Strongly Disagree<br>( ) Disagree<br>( ) Neutral<br>( ) Agree<br>( ) Strongly Agree
The videos provided by the PREP team helped new PREP team members successfully construct their own PREP Google quizzes.	( ) Strongly Disagree<br>( ) Disagree<br>( ) Neutral<br>( ) Agree<br>( ) Strongly Agree
**Questions About the Mentor-Mentee Relationship**	
The mentorship provided by second year medical student PREP team members helped new PREP team members understand expectations of team members.	( ) Strongly Disagree<br>( ) Disagree<br>( ) Neutral<br>( ) Agree<br>( ) Strongly Agree
The mentorship provided by second year medical student PREP team members helped new PREP team members understand the process to write PREP-style formative questions.	( ) Strongly Disagree<br>( ) Disagree<br>( ) Neutral<br>( ) Agree<br>( ) Strongly Agree
The mentorship provided by second year medical student PREP team members helped new PREP team members successfully write PREP-style questions.	( ) Strongly Disagree<br>( ) Disagree<br>( ) Neutral<br>( ) Agree<br>( ) Strongly Agree
**Questions About All Resources**	
Overall, I would rate (1-10 scale) the help provided by the second year PREP team members as:	( ) 1 (lowest quality)<br>( ) 2<br>( ) 3<br>( ) 4<br>( ) 5<br>( ) 6<br>( ) 7<br>( ) 8<br>( ) 9<br>( ) 10 (highest quality)
